# Barriers and Facilitators to Using Campus-Based Mental Health Services: An Exploratory Qualitative Study of University Students in Astana, Kazakhstan

**DOI:** 10.3390/healthcare14213674

**Published:** 2026-10-29

**Authors:** Indira Seitimova, Maiya Taushanova, Yerbol Nurkatov, Daniel Cavanagh, Kuanysh A. Yergaliyev

**Affiliations:** 1 Department of Biomedical Sciences, Nazarbayev University School of Medicine, 5/1 Kerey and Zhanibek Khandar Street, Astana 010000, Kazakhstan; indira.seitimova@alumni.nu.edu.kz (I.S.); yerbol.nurkatov@nu.edu.kz (Y.N.); 2 Public Health and Health Care Department, West Kazakhstan Marat Ospanov Medical University, Aktobe 030019, Kazakhstan; dr.taushanova@zkmu.kz; 3 Centre for Mental Health and Community Wellbeing, Melbourne School of Population and Global Health, University of Melbourne, Melbourne, VIC 3010, Australia; daniel.cavanagh@unimelb.edu.au; 4 School of Social Sciences, Business and Law, SDU University, Kaskelen 040900, Kazakhstan

**Keywords:** Kazakhstan, mental health services, mental well-being, on-campus counselling, university students

## Abstract

Background/Objectives: The mental health difficulties of youth in Central Asia are of increasing concern. This exploratory qualitative study explores university students’ accounts of barriers to and facilitators of using campus-based mental health services, their perceptions of accessibility and quality, and their suggestions for service improvement. Methods: Semi-structured interviews were conducted with 11 undergraduate students who responded to open invitations and studied at five universities (two private and three public) in Astana, Kazakhstan. Participants fell into three mutually exclusive groups: students who had used campus services (*n* = 6), students who had attempted to use them but did not receive a service (*n* = 2), and students who were aware of the services but had never attempted to use them (*n* = 3). The data were analyzed using codebook thematic analysis with a hybrid deductive–inductive approach. Results: Five themes were developed: personal triggers for seeking help; accessibility and institutional barriers; cultural and societal influences; experiences of service quality; and participants’ suggestions for improvement. Participants described experienced barriers (e.g., appointment delays, restricted opening hours and phone-only booking), perceived barriers (e.g., limited visibility of services and predominantly Russian-language provision) and anticipated barriers (e.g., family stigma and fear of psychiatric registration). Assessments of service quality came primarily from the six service users and centered on transparency about counsellors’ approaches and on session length. Participants suggested increasing service visibility, offering more flexible booking and opening hours, providing information about counsellors’ qualifications and approaches, offering support in more than one language, and reducing stigma through culturally sensitive education. Conclusions: Strengthening campus mental health services requires addressing institutional and sociocultural barriers to help-seeking. These findings are exploratory and specific to the studied context. Therefore, further research across diverse settings is needed to explore the transferability of these findings.

## 1. Introduction

Globally, university students usually attend university during a developmental period associated with heightened vulnerability to mental health problems. Approximately 75% of lifetime mental disorders begin by the age of 24, and approximately 35% of university students meet the criteria for a diagnosable mental health disorder [1,2,3]. Emerging research suggests there is an increasing number of young people reporting mental health difficulties in Central Asia [4]. Research conducted at one of the universities in Astana, Kazakhstan found that 24% of students self-reported moderate to severe depressive symptoms, while 10% met the criteria for clinical depression [5]. Despite the high prevalence of mental illness, young people are the least likely to seek professional help [6,7]. The reasons for low service utilization are extensively studied in Western contexts, with many studies pointing to stigma, a preference for self-reliant care strategies and poor mental health literacy [6,8,9]. However, the utilization and perceived quality of campus-based mental health services in Kazakhstan remain insufficiently explored [5,10].

Like other countries in Central Asia, the use of mental health services in Kazakhstan may be shaped in part by the legacy of the centrally planned, Semashko-style structure of the Soviet healthcare system [11]. During the Soviet period, psychiatry was biologically oriented, with most care taking place in asylum-like psychiatric hospitals [4], and psychiatry was at times used as ‘an instrument of power and control’ against political dissidents [12]. Studies of Russian-speaking immigrants and of adults in Russia suggest that this history may be associated with low trust in mental health professionals [12,13]; however, these findings cannot be assumed to apply directly to contemporary Kazakhstan, and Kazakhstan-specific evidence on trust in mental health services remains scarce. We therefore treat this history as a possible contextual influence rather than an established explanation. Limited participation of service users in policy development and decision making has also been reported across the region [4]. Stigma may also influence the use of mental health services among university students in Central Asia. Mental health stigma represents a significant barrier to help-seeking, as psychological difficulties are frequently misunderstood, minimized, or regarded as taboo [5,14,15]. Population studies indicate that public knowledge, beliefs and attitudes about mental illness vary considerably across settings [16,17]. Although research on stigma in Central Asia is limited, a regional review has described the registration of psychiatric diagnoses as a factor that may amplify stigma through perceived consequences for marriage and employment [4]. Campus-based psychological counselling, which does not usually involve psychiatric diagnosis, should be distinguished from psychiatric diagnosis and registration within the health system [18,19]. Whether or not such consequences apply to counselling, fear of registration may be of particular concern to university students who are seeking employment or are considering marriage.

In Kazakhstan, psychiatric registration (referred to as “dynamic monitoring”) is instituted exclusively by specialized psychiatric services (Mental Health Centers) and requires an official decision by a medical advisory commission, rather than the unilateral decision of an individual physician [19]. Regarding the diagnoses subject to such monitoring, the list is strictly limited to severe, persistent, chronic, or frequently relapsing mental disorders with pronounced clinical manifestations. The mere fact of being under dynamic observation does not automatically prohibit employment, nor does it prevent marriage. There are only specific restrictions regarding the performance of certain types of activities [20,21]. Psychological support services at Kazakhstani universities and colleges (campus psychologists) provide psychological, educational, and counseling assistance. These services are not psychiatric medical facilities; they do not have access to the unified medical information system used by psychiatric centers and do not transmit data to Mental Health Centers. Consequently, consulting a university (campus) psychologist cannot, under any circumstances, result in being placed on the psychiatric registry or under dynamic monitoring.

There is a growing understanding of the need to better understand the mental health difficulties of young people in Central Asia [22]. While the lack of community-based and psycho-social services, overreliance on hospital-based psychiatric treatment and general insufficient resources dedicated to mental health services are shared throughout the region, there are important distinctions between each of the countries [11,22]. For example, Kazakhstan previously reported one of the highest youth suicide rates in the world [23]. Since this time, the Government of Kazakhstan has rolled out Psychological Support Centers across the country, launched a multisectoral program on suicide prevention and has begun the development of a national Concept on Mental Health [24], all of which may influence help-seeking in distinct ways. As such, differences in cultural norms, institutional arrangements, and social attitudes toward psychological support may shape help-seeking behavior in Kazakhstan differently from patterns commonly described in Western settings, while reflecting the broader historical and structural context of mental health care in Central Asia [11,15,22].

The study was conducted among students from five universities in Astana city, including two private and three public institutions. Astana, as the capital and a major educational and administrative center of Kazakhstan, attracts students from diverse socioeconomic backgrounds and geographical origins. This location provided an opportunity to capture a range of experiences of university-based mental health services across different institutional contexts. The inclusion of both public and private universities, which also differed in their counselling structures and resource allocation, allowed the study to capture a range of institutional settings, although the study was not designed to compare sectors. However, Astana represents a predominantly urban and comparatively well-resourced context and may differ from smaller cities and rural areas of Kazakhstan in terms of socioeconomic conditions, migration patterns, availability of health services, and proximity to family and community support. Therefore, the findings should be interpreted within the specific context of Astana and should not be assumed to be representative of university students across Kazakhstan. Accordingly, the aim of this study was to explore university students’ experiences with mental health services provided within higher education institutions in Astana, Kazakhstan including their perceptions of how well these services respond to students’ needs. Specifically, the study sought to address the following research questions: (1) What do students describe as barriers to and facilitators of using campus-based mental health services?; (2) How do students perceive the accessibility and quality of available mental health support services? (3) What recommendations can be derived from students’ experiences to enhance university mental health services?

## 2. Materials and Methods

### 2.1. Study Design

This study adopted a qualitative descriptive design, informed by a theoretical framework (Section 2.2), to explore university students’ experiences with campus-based mental health services, including service utilization, perceived accessibility and quality, and recommendations for improvement. A qualitative approach was selected to allow for an in-depth exploration of participants’ perspectives and meanings, which are particularly relevant for understanding complex, context-dependent phenomena such as help-seeking behavior and perceptions of mental health care [25]. The study was conducted from an interpretivist epistemological stance, whereby participants’ accounts were understood as situated descriptions of their experiences and perceptions, and the findings represent the researchers’ interpretation of these accounts rather than an objective assessment of campus mental health services.

### 2.2. Participants and Sampling

Participants were recruited using purposive sampling strategy to ensure the inclusion of individuals with relevant knowledge and experience of university mental health services [26]. Recruitment invitations were distributed by the first author through student group chats on WhatsApp and Telegram and through social media platforms across the five participating universities. University staff and administrators were not involved in identifying or referring participants and did not act as recruitment gatekeepers. No financial or other compensation was offered for participation. Students who expressed interest were contacted by the first author, given further information, and, if eligible, scheduled for an interview. Eligibility criteria included: (1) enrollment in an undergraduate program; (2) age between 18 and 24 years; and (3) experience with or familiarity with on-campus mental health services, either through direct use or awareness of their availability. At the sample level, purposive sampling additionally sought variation in gender, academic year, field of study and institution type; this was a sampling objective rather than an individual eligibility criterion. Awareness of campus services was required because the study aimed to examine students’ experiences and perceptions of existing services; as a consequence, students who were entirely unaware of these services could not take part, which we acknowledge as a major limitation (Section 5). Participants were classified into three mutually exclusive groups according to their highest level of engagement with campus services: students who had used campus services (*n* = 6), students who had attempted to use them but did not receive a service (*n* = 2), and students who were aware of the services but had never attempted to use them (*n* = 3). Of the 15 students who initially expressed interest, 2 did not proceed to eligibility screening. The remaining 13 students were screened and all met the eligibility criteria. Of these 13 eligible students, 11 completed an interview, while 2 did not complete the interview process. Available recruitment records did not permit further differentiation of the reasons for non-participation. The stopping point was decided by the first author (I.S.) in consultation with the supervising senior authors (K.A.Y., Y.N.) through review of new codes after each interview. We aimed for code saturation, which was assessed by the first author and the supervisors through ongoing review of the codes generated across interviews. Recruitment ceased when review of successive interviews indicated that no substantively new codes were emerging from the data. Because participants came from five institutions, with one to four participants each, saturation was assessed for the sample as a whole and not within institutions. Information power [27] was used as a complementary rationale: a relatively modest sample was considered adequate given the specific aim, the sample’s direct relevance to that aim, the use of an established theoretical framework, and the information-rich nature of the interview dialogue. The composition of the final sample is detailed in Table 1.

### 2.3. Theoretical Framework

The study was guided by the Modified Mental Health Intervention Spectrum (MMHIS), originally proposed by Mrazek and Haggerty and subsequently adapted by Barry et al. [28]. The framework conceptualizes mental health interventions across three interconnected phases: prevention, treatment, and maintenance, and emphasizes four key domains: resilience, supportive environments, empowerment, and competence. These components informed the development of the semi-structured interview guide. Questions addressing resilience explored how students manage stress and maintain their well-being; supportive environments examined campus resources, social networks, and peer support; empowerment explored students’ ability to recognize their mental health needs and seek and access appropriate services; and competence addressed students’ knowledge and capacity to manage mental health needs. Questions related to prevention, treatment, and maintenance explored awareness of preventive resources, experiences with available mental health services, and access to ongoing support. The framework was used in three ways: (1) to structure the interview guide; (2) to provide an initial set of deductive codes during analysis; and (3) to interpret the final themes (Table 2). It did not inform sampling. The analysis therefore used a hybrid deductive–inductive approach. Initial deductive codes were informed by the MMHIS while additional codes were generated inductively from participants’ accounts when relevant content was not adequately captured by the framework. Examples of inductively generated codes included language of service provision and appointment scheduling. This approach allowed issues emerging from participants’ accounts to be retained rather than restricting the analysis to the predefined framework.

### 2.4. Setting

The study was conducted in five universities in Astana, the administrative and political capital of Kazakhstan. The selected universities differed in institutional size, student population, organizational structure, and approaches to mental health support. Including both public and private universities enabled an exploration of potential disparities in mental health service accessibility and utilization across institutional contexts. They also varied in the availability and organization of counselling services, including differences in mental health professionals, referral pathways, and resources available to students. These institutional differences allowed the study to capture a range of student experiences and examine how variations in university structures and service provision may influence access to and utilization of mental health support. Institutional characteristics were used as sampling parameters only; they were not independently audited or verified, and any institution-level differences reported in this study should be understood as participants’ perceptions rather than as objective measures of service provision.

### 2.5. Data Collection

Data were collected through individual semi-structured, in-depth interviews conducted between late January and March 2025. Interviews ranged from 30 to 60 min in duration. All interviews were conducted by the first author (I.S., MPH), a female researcher trained in qualitative interviewing, under the supervision of the senior authors (K.A.Y., Y.N.), who provided ongoing debriefing. The interviewer had no prior relationship with the participants, who were recruited through open invitations. An interview guide comprising ten open-ended questions was developed based on a review of relevant literature and informed by the Modified Mental Health Intervention Spectrum. The guide was organized into seven thematic sections: (a) participant background; (b) overview of available mental health services; (c) service utilization; (d) perceptions of accessibility and quality; (e) cultural and social influences; (f) supportive environments and recommendations for improvement; and (g) concluding reflections. The guide was pilot-tested before the main data collection with one university student with characteristics similar to the target population. The pilot interview was conducted in Russian. Based on the pilot interview, additional probes were added to facilitate more detailed responses. The pilot participant met the study eligibility criteria, and the interview was included in the final analytic sample because the core questions and interview domains remained unchanged. It was prepared in Russian, Kazakh, and English, and each interview was conducted in the participant’s preferred language (*n* = 2 Kazakh; *n* = 9 Russian). The interview guide can be found in Supplementary Materials S1.

Interviews were conducted either face-to-face (*n* = 5) in private rooms on university campuses or online via the Zoom platform (*n* = 6), according to participants’ preferences. The same interview guide and probing strategy were used in both modes. Participants interviewed via Zoom were asked to join from a private location where they could not be overheard, and use of the camera was optional. Differences in interview length, depth, or participant disclosure between face-to-face and Zoom interviews were not formally assessed. All interviews were audio-recorded with verbal informed consent and transcribed verbatim. Interviews conducted in Kazakh or Russian were translated into English using Google Translate, and subsequently reviewed and corrected by the first author against the original-language transcripts to ensure accuracy and consistency before qualitative analysis. Transcripts were de-identified to protect participant confidentiality before analysis.

### 2.6. Data Analysis

Data were analyzed using codebook thematic analysis, as distinguished by Braun and Clarke [29,30], from both coding-reliability and reflexive approaches: a structured codebook is developed and applied to the data, and some themes are anticipated before full analysis. We used a hybrid deductive–inductive approach [31]. An initial set of codes was derived deductively from the domains of the Modified Mental Health Intervention Spectrum and the interview guide, and further codes were generated inductively from the transcripts during coding. This approach was selected, rather than reflexive thematic analysis, because the study used an a priori organizing framework and a structured codebook applied by more than one coder.

The analytic process involved: (1) familiarization with the data through repeated reading of transcripts; (2) generation of initial codes combining framework-derived categories with inductively generated codes; (3) development and iterative refinement of a codebook including code definitions and exemplar excerpts; (4) construction of candidate themes through the organization of related codes; (5) review and refinement of themes to ensure coherence and distinction; and (6) definition and naming of final themes. Coding was predominantly semantic, and codes were applied to meaning units (a sentence or group of sentences expressing a single idea). Themes were constructed as patterns of shared meaning across participants rather than as summaries of interview topics. Coding was carried out by more than one member of the research team (I.S., Y.N. and K.A.Y.), and coding decisions and discrepancies were discussed and resolved through team consensus, supported by peer debriefing with the supervisor. A detailed description of the codebook can be found in Supplementary Materials S2. Participants were coded to preserve confidentiality using identifiers such as PW (Participant Woman) or PM (Participant Man), followed by their year of study, university (e.g., private 1, public 2) and service-use status (U = used services; A = attempted; N = aware but never attempted). NVivo qualitative data analysis software was used to facilitate data organization and coding. Reflexivity was maintained throughout the analytic process through memo-writing and ongoing reflection on the researcher’s positionality and assumptions [30]. The first author, who conducted the interviews, is a female, MPH-trained public health researcher familiar with the university context and student mental health. The research team discussed her positionality, prior assumptions, and relationship to the topic and considered their potential influence on data collection, coding, and interpretation. Reflexive discussions were used to identify and challenge these assumptions.

To enhance methodological rigor, the study followed established criteria for trustworthiness in qualitative research, including credibility, dependability, confirmability and transferability [32]. Credibility was supported through the use of verbatim transcription and systematic analytic procedures, and peer debriefing with the supervisors. An audit trail documenting analytic decisions and theme development was maintained to enhance dependability. Confirmability was supported through researcher reflexivity and documentation of assumptions. Detailed descriptions of the research context, participants, and analytic process were provided to support transferability and allow readers to assess the applicability of findings. Reporting follows the COREQ checklist [33] and the completed checklist is provided in Supplementary Material S3. No repeat interviews were conducted, field notes were taken, transcripts were not returned to participants, and member checking and participant feedback on the findings were not undertaken.

Ethical approval for the study was obtained from the Institutional Research Ethics Committee (IREC) on 15 January 2025 (reference number: 2024Nov#12). All procedures were conducted in accordance with ethical standards for research involving human participants. The researchers completed certification through the Collaborative Institutional Training Initiative (CITI) in research ethics and human subjects protection. Prior to participation, verbal informed consent was obtained from all participants. Verbal consent was preferred to protect participants’ confidentiality since the study could have been perceived as sensitive by young people. Participants’ consent was audio-recorded at the beginning of each interview and documented by the researcher in the study records. Participants were informed that participation was voluntary, that confidentiality would be maintained, and that they could withdraw from the study at any time without penalty. Potential risks, including mild emotional discomfort, were explained, and participants were provided with contact information for university psychological support services. All identifying information was removed during transcription, and audio recordings and transcripts were securely stored.

## 3. Results

A total of 11 students were recruited from five universities in Astana, Kazakhstan, including two private and three public institutions. Sociodemographic characteristics of the participants are presented in Table 1.

**Table 1:** Sociodemographic Characteristics of the Participants.

Variable	Category	n	%
Gender	Female	8	72.7
	Male	3	27.3
Year of study	Freshman (1st year)	1	9.1
	Sophomore (2nd year)	3	27.3
	Junior (3rd year)	3	27.3
	Senior (4th year)	4	36.4
Institution type	Private	6	54.5
	Public	5	45.5
Age	Median 23 (range 18–24) years	11	–
Field of study	Management	2	18.2
	Sinology	1	9.1
	History	1	9.1
	Biology	2	18.2
	Medicine	1	9.1
	Finance	1	9.1
	Journalism	1	9.1
	Transport and Power Engineering	1	9.1
	Environmental engineering	1	9.1
University distribution	Private university [1]	4	36.4
	Private university [2]	2	18.2
	Public university [1]	2	18.2
	Public university [2]	2	18.2
	Public university [3]	1	9.1
Service-use status	Used services	6	54.5
	Attempted, did not receive a service	2	18.2
	Aware, never attempted	3	27.3
Interview language	Russian	9	81.8
	Kazakh	2	18.2
Interview mode	Face-to-face	5	45.5
	Zoom	6	54.5
Residence	Dormitory	4	36.4
	Off-campus	7	63.6

Note. Percentages are rounded to one decimal place and may not sum to 100. Service-use categories are mutually exclusive and reflect each participant’s highest level of engagement with campus services. Additional characteristics are reported as far as confidentiality allows given the small sample.

**Figure 1:**
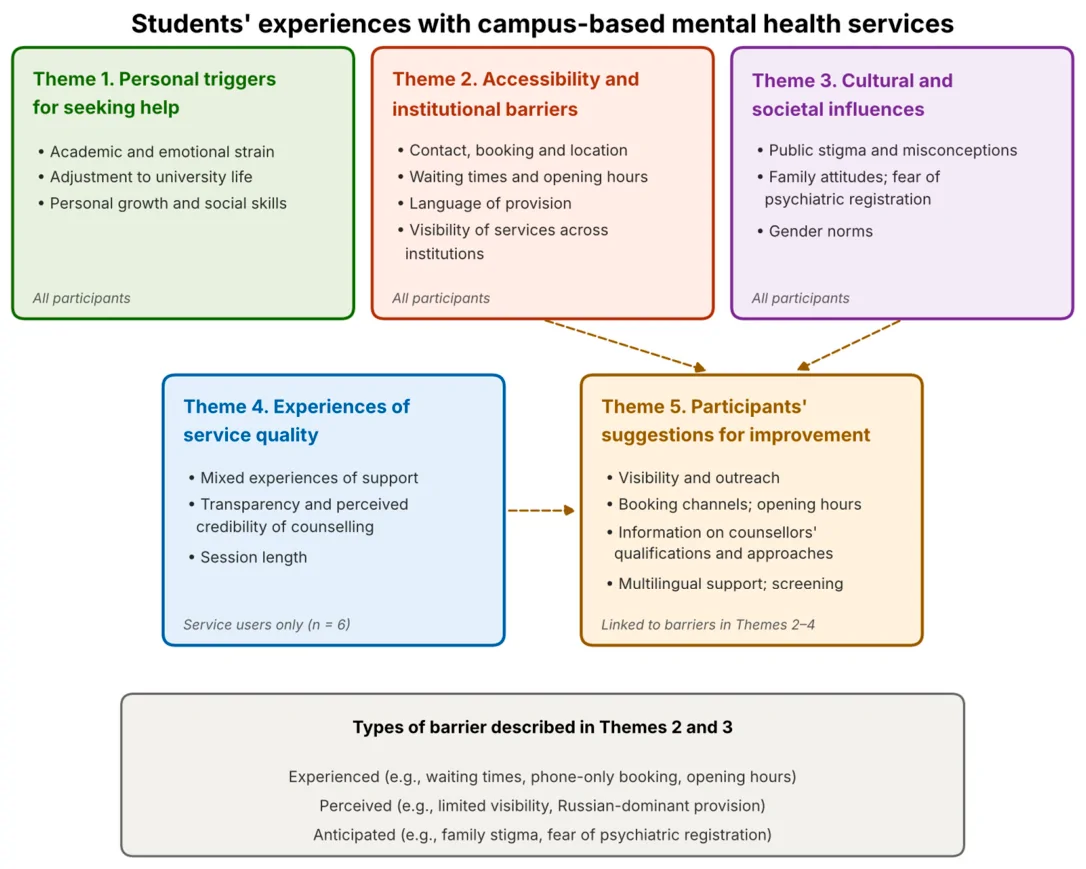
Thematic map showing the five themes and their subthemes. Dashed arrows indicate that participants’ suggestions (Theme 5) respond to barriers described in Themes 2–4. The themes are interrelated and are not presented as a causal sequence.

The analysis yielded five themes, which are shown with their subthemes in Figure 1: (1) personal triggers for seeking help; (2) accessibility and institutional barriers; (3) cultural and societal influences; (4) experiences of service quality; and (5) participants’ suggestions for improvement. To keep the themes conceptually distinct, Theme 2 covers the institutional conditions that facilitated or constrained access (contact and booking arrangements, waiting times, opening hours, location, visibility and language of provision), as described by all participants, whether these conditions were experienced directly, perceived, or anticipated; Theme 4 is restricted to evaluations of actual encounters with counsellors by the six participants who had used services; and Theme 5 reports participants’ own suggestions, each linked to the barrier it addresses rather than describing it again. Each quotation is presented as evidence for one theme only. Frequencies are reported as the number of participants (out of 11, or out of 6 service users for Theme 4) whose accounts were coded to a theme or subtheme. Table 2 shows how each theme relates to the domains and phases of the Modified Mental Health Intervention Spectrum. In this mapping, ‘competence’ refers only to students’ own capacity to recognize and respond to distress, and participants’ views of counsellors are mapped to the treatment phase rather than to the competence domain.

**Table 2:** Relationship between themes and the Modified Mental Health Intervention Spectrum (MMHIS).

Theme	Subthemes	MMHIS Domain	MMHIS Phase	Evidence Base
1. Personal triggers for seeking help	Academic and emotional strain/adjustment to university life; personal growth and social skills.	Competence (students’ capacity to recognize distress); resilience	Entry into treatment	All participants
2. Accessibility and institutional barriers	Contact, booking and location; waiting times and opening hours; language of provision; visibility across institutions.	Supportive environments	Prevention; treatment (access)	All participants (experienced, perceived and anticipated barriers)
3. Cultural and societal influences	Public stigma and misconceptions; family attitudes and fear of registration; gender norms.	Empowerment (constraints on students’ agency)	Prevention	All participants
4. Experiences of service quality	Transparency and perceived credibility of counselling; mixed experiences of support; session length.	— (provider side; not mapped to a domain)	Treatment; maintenance (session limits)	Service users only (n = 6)
5. Participants’ suggestions for improvement	Visibility and outreach; booking channels; opening hours; information on counsellors; multilingual support; screening.	Supportive environments; empowerment	Prevention; treatment; maintenance	Participants’ suggestions (not evaluated interventions)

### 3.1. Personal Triggers for Seeking Help

(a) Academic and emotional strain/Adjustment to university life

The results showed that 7 of 11 participants described recognizing their own psychological difficulties as what prompted them to seek, or consider seeking, help. They described emotional and academic difficulties, as well as challenges adapting to university life.

*“I thought it’s hard for me. I left my comfort zone… I developed psychological issues—self-perception, social difficulties…I thought about calling, but there was no connection…”*

*[PW1, senior, private 1]*

*“My grades became very bad… my sleep was disrupted… You’re far from home, existing on your own”*

*[PW3, senior, private 1]*

(b) Personal growth and social skills

Beyond immediate mental health challenges, 3 participants described seeking professional support for self-discovery, personal growth, and improving their communication skills.

*“I had difficulties interacting with society. In our academic environment, social interaction is essential, which is why I decided to seek help.”*

*[PM9, senior, public 1]*

### 3.2. Accessibility and Institutional Barriers

This theme describes the institutional conditions that facilitated or constrained access to campus services. It includes conditions that participants had experienced when trying to use services, as well as conditions they perceived or anticipated without having attempted to use them; this distinction is indicated in the text.

(a) Contact, booking and location

Six participants described services as easy to contact. Multiple contact options, such as WhatsApp and hotlines, made it easier to seek help, and 4 participants noted that services were free of charge.

*“You can just write to WhatsApp or call, and you can just come at any time…”*

*[PM2, sophomore, public 1]*

*“…Well, I know that they are available, that there is some kind of hotline, that it is free, you can make an appointment”*

*[PW4, sophomore, private 1]*

*“You can call a psychologist for urgent support, and there is also a psychologist’s office where you can schedule an appointment in advance. It’s free…Students have the option to switch their psychologist.”*

*[PW1, senior, private 1]*

Five participants described the location of services near dormitories as making support easier to reach.

*“Our university is well structured, easy access… you can get help quickly.”*

*[PW1, senior, private 1]*

*“We have psychological support at the university… it is located not far from the dormitory, very convenient.”*

*[PM9, senior, public 1]*

In contrast, one participant, a junior student from a private university, explained that she postponed making an appointment for a year because booking required a phone call. Her account suggests that offering booking channels other than the telephone (e.g., messaging apps) may matter for some students.

*“You have to call to make an appointment… because of that, I spent a year going without signing up.”*

*[PW3, junior, private 1]*

(b) Waiting times and opening hours

Three participants described having to wait for a response from the call centre or for an allocated appointment date. They felt that problems could become outdated or worsen in the meantime, and 2 reported turning to paid psychological help off campus. One sophomore student from a private university explained that the lack of an urgent appointment led her to seek help elsewhere:

*“At university, they signed me up for a certain date; there was no urgent appointment. I think I first contacted a private one, because she was ready to accept me earlier than on campus.”*

*[PW4, sophomore, private 1]*

Another participant explained that the expected one-month wait made contacting a psychologist seem pointless:

*“Since in any case the next appointment will be in a month, I don’t see any point in contacting a psychologist with an outdated problem.”*

*[PW6, junior, private 2]*

Four participants also described opening hours as a barrier, because services were available only during the hours when they had classes:

*“The working hours are from Monday to Friday, 9 AM to 6 PM, but our classes usually end around 6 PM…we can’t visit a psychologist during our free time.”*

*[PW5, senior, public 3]*

(c) Language of provision

Two participants described the language of provision as a barrier. They noted that not all Kazakhstani students are bilingual and that international students who do not speak Russian or Kazakh may struggle to receive support. These accounts reflect participants’ perceptions and were not verified against service records.

One sophomore student mentioned the lack of Kazakh or English-speaking professionals.

*“We had one or two Kazakh speaking specialists, the rest were limited to Russian, so I understand that specialists in Astana can only be specialists in the Russian language, but we have Chinese Kazakhs and foreign students. No one can say for sure whether specialists know English or not. Those who know Russian have more chances to get help.”*

*[PM11, sophomore, private 1]*

(d) Visibility of services across institutions

Accounts of how visible services were differed between institutions. 3 participants from Private University 1 described services as well promoted, and one of them expressed the view that her university invested more effort in mental health provision than public universities. These are institution-specific accounts by individual participants; they were not independently verified and do not support conclusions about private or public universities as sectors.

*“Our university puts in more effort in this area compared to public ones. Universities should take it as an example, though I am not saying that our university has reached its full potential…”*

*[PW1, senior, private 1]*

Participants from Private University 1 described posters in campus facilities and spaces for both social interaction and privacy:

*“In every restroom there are posters saying: ‘If you have problems… You can call anytime’”*

*[PW1, senior, private 1]*

*“Unlike other universities, we have a modern interior, all the necessary infrastructure, and plenty of spaces where you can interact with people and where you can be alone.”*

*[PW4, sophomore, private 1]*

In contrast, 3 of the 5 participants from public universities described learning about services only through posters in dormitories, or not knowing about them at all:

*“I studied for four years and never knew we had psychologists… Only saw posters in the dormitory”*

*[PM9, senior, public 1]*

### 3.3. Cultural and Societal Influences

(a) Public stigma and misconceptions

A total of 10 participants described stigmatising ideas about mental health services held by their relatives, peers and wider society. Participants felt that poor understanding of terms such as psychology, psychotherapy and therapy contributed to these services being undervalued. They reported that common stereotypes linked mental health care to taboo topics, mental illness and even “craziness”.

*“I don’t think our society is ready to accept it yet. Not psychological services, they wouldn’t even be prepared to respect the personal boundaries of other people…” You couldn’t handle your own problems, and what’s so bad? Are you weak? …”That’s a kind of criticism, so it’s very critical in our society.”*

*[PW5, senior, public 3]*

However, this view was not shared by all participants. One participant described a contrasting perception, suggesting that psychological help had become more socially acceptable among younger people:

*“I think not. Nowadays, young people are already open to it. Most young people do not see anything shameful about going to a psychologist, so I don’t think society or culture has much influence.”*

*[PW8, junior, public]*

(b) Family attitudes; fear from psychiatric registration

Seven participants described their families’ concerns about, and misunderstanding of, psychological services. For example, a junior student from a private university described her mother’s fear that she could be hospitalized or put on the registry as mentally ill; this was an anticipated consequence rather than one the participant had experienced.

*“At first, she [the mother] thought: “What if they put you on the register there?”. She thought it was something unserious, even the very word stress and depression was not perceived there as a real, existing thing.”*

*[PW3, junior, private 1]*

Another participant described a lack of acceptance of psychological support among older people:

*“The older generation doesn’t recognize psychologists at all. For them, it’s only for psychos.”*

*[PM11, sophomore, private 1]*

(c) Gender-Related Barriers

Two participants (including 2 of the 3 men) described masculine norms as making men reluctant to seek help. They described expectations that men should suppress their emotions and avoid showing vulnerability, which they felt made it difficult for men to seek external support. One sophomore student described how difficult it is for men to share emotions even within close relationships, let alone with external mental health professionals.

*“[…] men in general are forced to have their emotions there. There is a slogan that you have to be a man, strong and so on… it is difficult to share problems, even with close people, let alone external specialists.”*

*[PW4, sophomore, private 1]*

### 3.4. Experiences of Service Quality

This theme is based only on the accounts of the six participants who had used campus services. Descriptions of counsellors reflect participants’ perceptions of their encounters and are not an assessment of counsellors’ actual qualifications or competence.

(a) Transparency and perceived credibility of counselling

Four of the six service users described encounters that left them uncertain whether the counsellor had understood their concerns, and 3 felt uninformed about the methods being used.

*“He asked, “What’s your problem?” after I shared everything; it mixed sadness with anger.”*

*[PM2, sophomore, public 1]*

*“They didn’t specify what approach they were using… there was a lack of information”*

*[PM9, senior, public 1]*

(b) Mixed experiences of support

Service users described varied experiences of professional help on campus. 4 of the 6 service users described positive experiences, emphasizing the supportive nature of face-to-face sessions and group workshops and the importance of a comfortable environment in which they felt encouraged to speak up.

*“They created an environment where it didn’t feel like an official meeting but more like an informal gathering, allowing everyone to feel at ease… Everyone could freely express themselves. Even those who were slightly socially anxious or introverted…”*

*[PW10, freshman, public 2]*

In contrast, 2 service users felt that the techniques offered did not fit their concerns. One participant found the suggested exercise too simple for her situation, and another did not understand the purpose of a specific technique.

*“The techniques felt vague; after all I shared, I got a simple meditation exercise.”*

*[PW4, sophomore, private 1]*

*“There was some technique with cards… But honestly, I didn’t understand it. I didn’t draw any conclusions from it.”*

*[PM2, sophomore, public 1]*

(c) Session length

Two service users found that the strict one-hour duration and limited number of sessions hindered their ability to fully engage in meaningful discussions. One participant expressed frustration that the time constraints often disrupted the flow of conversation, particularly when deeper issues began to surface.

*“There are limits on visiting a psychologist… strictly limited sessions to one hour, even interrupting when deeper conversations began…within this time, you are expected to share all your problems, receive feedback… This rigid time constraint made it difficult to open up, and by the time I felt comfortable, the session would end.”*

*[PM11, sophomore, private 1]*

### 3.5. Participants’ Suggestions for Improvement

This theme reports suggestions made by participants themselves. Each suggestion is linked to the barrier it responds to in Section 3.2, Section 3.3 and Section 3.4, and those barriers are not described again here. The authors’ interpretation of these suggestions is presented in Section 4.3.

(a) Awareness and visibility

In response to the uneven visibility described in Section 3.2, 4 participants suggested promoting mental health services across the whole campus so that all students, not only those living in dormitories, are informed. One sophomore student from a public university noted that he had seen information about services only in the dormitory:

*“I would never have known if I didn’t live in a dorm.”*

*[PM2, sophomore, public 1]*

Six participants suggested that organizing regular workshops and seminars could help reduce stigma and encourage students to seek help. One freshman student felt that such activities can foster open communication and empower students to speak up, even if they initially feel embarrassed.

*“Workshops or stress-relief weeks can encourage students to share openly.”*

*[PW3, junior, private 1]*

*“Every week some mental workshops help to unite classmates, new acquaintances, not everyone talks about it, because everyone is embarrassed. But this, on the contrary, will help students to open up even more in a positive way.”*

*[PW10, freshman, public 2]*

(b) Booking channels; opening hours

In response to the booking difficulties described in Section 3.2, 2 participants suggested digital solutions such as chatbots or apps for scheduling.

*“It would be better to use a bot or app for appointments.”*

*[PW4, sophomore, private 1]*

In addition, one senior student suggested that psychological screening during admission could help identify students’ mental health needs early on.

*“Psychological screening at admission could minimize students’ psychological problems.”*

*[PW1, senior, private 1]*

In response to the opening hours described in Section 3.2, 3 participants suggested extending service hours beyond the end of classes.

*“The psychologist is available only from 9 am to 6 pm. If they worked longer, more students with busy schedules could book appointments.”*

*[PW10, freshman, public 2]*

(c) Information about counsellors’ qualifications and approaches

In response to the uncertainty described in Section 3.4, 3 participants suggested that services provide information about counsellors’ qualifications and therapeutic approaches. One junior student from a private university explained:

*“I’m not sure about the quality of their work or their qualifications, or how much they are actually able to help.”*

*[PW7, junior, private 2]*

(d) Multilingual support

In response to the language barriers described in Section 3.2, 2 participants suggested offering psychological support in more than one language. A senior student from a private university felt that mutual understanding is essential for effective communication between students and professionals.

*“Understanding each other is essential; otherwise, conversations are useless.”*

*[PW1, senior, private 1]*

## 4. Discussion

### 4.1. Factors Shaping Students’ Utilization of Mental Health Services (RQ1)

Participants’ accounts suggest that help-seeking was shaped by the interaction of psychological distress, institutional organization, and sociocultural norms. Emotional strain related to academic pressure and adjustment difficulties was described as the main reason for seeking support, whereas participant use of services appeared to depend on how accessible the services were, as well as on cultural perceptions of mental health. These accounts suggest that help-seeking in the university context of Astana is not solely an individual response to distress, but a process embedded within institutional and societal contexts [2,3,34].

Participants described institutional conditions as both facilitating and constraining service use. They highlighted the importance of user-friendly appointment systems, including messaging platforms such as Telegram or WhatsApp, as well as the visibility of services through posters, workshops, and awareness campaigns. The proximity of counselling services to dormitories was also perceived as facilitating engagement. These accounts are consistent with evidence that logistical convenience and digital accessibility are associated with student engagement with psychological support services [35,36,37].

Conversely, long waiting times, limited availability of urgent care, and inflexible service hours were described as barriers to help-seeking. Some participants reported turning to private, fee-based services when university counseling was unavailable or delayed. Structural constraints such as administrative inefficiencies and limited service capacity have been widely recognized as factors that impacts trust and continuity of care [15,36,37,38]. The continued reliance on phone-based appointment systems was described as discouraging by one participant, which is consistent with evidence that inefficient service structures may inhibit help-seeking behavior [20,35,36,37].

Cultural factors also featured in participants’ accounts of whether to seek support. Participants described traditional attitudes toward mental health, often voiced by family members and the wider community, as a barrier to help-seeking, as these attitudes are associated with shame, fear of judgment, and reluctance to appear vulnerable. These accounts mainly concern perceived public and family stigma; internalized stigma was not coded separately and is not claimed here. Perceived stigma is a well-established barrier to mental health service use [15], and participants’ accounts suggest that it was salient for students in this sample. Participants described misunderstandings of mental health and therapy, particularly among older generations, which they felt led to psychological difficulties being minimized or stigmatized; similar barriers have been reported among students in other settings [39,40], although Kazakhstan-specific evidence remains limited. Finally, one participant described her mother’s fear that seeking help could lead to psychiatric registration. This fear should be distinguished from the actual legal and institutional consequences of registration, which relate to psychiatric diagnoses rather than to campus counselling [19]; regardless of its accuracy, such a fear may discourage help-seeking.

Participants also described gender norms as discouraging help-seeking among male students. They described cultural expectations that encourage emotional restraint and self-reliance, discouraging men from acknowledging psychological distress or seeking professional support. The influence of masculine norms on delayed help-seeking reflects broader gendered expectations surrounding emotional expression, which participants described as salient in their cultural context [41]. Because only three men took part, this finding should be interpreted with particular caution.

Language barriers, although less frequently discussed, were described as an additional obstacle to service use. Participants perceived counselling services to be predominantly available in Russian, limiting access for students who primarily speak Kazakh or for international students. This finding echoes prior research calling for culturally and linguistically competent mental health services to reduce disengagement and ensure equitable access [36,42]. In a multilingual context such as Kazakhstan, language inclusivity may be important for effective communication and sustained engagement with mental health services.

Beyond confirming global patterns of stigma, structural barriers, and academic stress as drivers of help-seeking, participants’ accounts point to several features of mental health service use that may be specific to this context. First, some participants from public universities reported limited awareness of available psychological services even after several years of enrollment. Second, participants described intergenerational attitudes that may reflect historically rooted perceptions of psychiatric care, including parental fears of labeling and long-term social and economic consequences of being put on the registry (an anticipated rather than experienced consequence). Third, the linguistic centralization of counseling services in Russian was perceived as a barrier for Kazakh-speaking and international students. Together, these findings suggest that student engagement with mental health services in Kazakhstan is embedded not only in individual and logistical factors, but also in historically and institutionally shaped sociocultural dynamics. Importantly, these findings should be interpreted within the clinical and institutional landscape of Astana, a major urban and higher education center with a relatively concentrated network of universities and campus-based psychological support services. This context may differ from that of smaller cities and rural areas, where the availability and organization of student mental health services may be more limited. Nevertheless, the present findings suggest that the presence of services does not necessarily translate into meaningful access. Students described delays in obtaining appointments, limited urgent support, scheduling systems that were not always student-friendly, restricted opening hours, uneven visibility of services, and uncertainty about service quality. These barriers highlight that effective access depends not only on the existence of services, but also on their organization, communication, responsiveness, and perceived credibility. Thus, while Astana provides an important setting for examining student mental health service utilization, these findings should not be assumed to directly reflect experiences in less-resourced urban or rural settings across Kazakhstan.

### 4.2. Interpreting Students’ Perceptions of Accessibility and Quality of Mental Health Services (RQ2)

Students’ perceptions of accessibility were shaped not only by the physical presence of services on campus, but also by how easily those services could be accessed. Although counseling centers were geographically available within university settings, participants’ accounts suggest that practical engagement depended on scheduling flexibility and ease of booking. Digital appointment systems and visible service promotion were interpreted as signals of institutional responsiveness, whereas rigid schedules and limited urgent support reduced perceived usability. Accessibility, therefore, functioned less as a matter of physical proximity and more as an indicator of institutional attentiveness to student realities [34,35].

Perceptions of service quality, reported by the six participants who had used services, were varied. Positive experiences were associated with emotional safety and empathetic communication. However, uncertainty regarding counsellors’ qualifications and limited transparency about therapeutic approaches contributed to skepticism among some service users. In these accounts, trust was shaped by clarity of communication and perceived professional credibility. Because the study examined perceived quality and acceptability rather than effectiveness or clinical outcomes, these findings cannot indicate whether services improved students’ mental health; they suggest, however, that perceived quality depended strongly on relational dynamics and transparency.

Some participants perceived private-university services as more visible or responsive than public-university services. Because these impressions were volunteered by a minority, were not consistent across the sample, and were not independently verified, we interpret them cautiously as perceptions that may reflect differences in outreach or institutional culture rather than demonstrated differences in service quality or student need [42,43].

### 4.3. Implications of Student Recommendations for Improving Mental Health Services (RQ3)

Students’ recommendations suggest that improvements in university mental health services require both organizational adjustments and cultural change. Increasing visibility of counseling services through orientation programs, academic spaces, and digital platforms was described as a necessary step toward normalizing help-seeking behavior within the university environment. In contexts where stigma remains influential, proactive outreach strategies have been shown to facilitate service utilization [36], and participants’ accounts suggest that such measures may be particularly relevant in the Kazakhstani setting.

The MMHIS provides a useful framework for interpreting these implications. Improvements in service visibility, outreach, and mental health education may contribute to supportive environments and prevention by helping students recognize available sources of support before distress escalates. Student-friendly booking systems, extended service hours, and multilingual provision may promote empowerment by increasing students’ practical ability to act on recognized mental health needs. At the treatment level, uncertainty about counsellors’ approaches and qualifications suggest that access alone may be insufficient if students do not perceive services as credible, appropriate, and responsive. Finally, sustained engagement with support requires attention to maintenance, including whether students can access timely help and remain connected to appropriate care as their needs persist or change over time.

These implications differ in their evidential basis and feasibility. Increasing visibility, offering booking channels other than the telephone, extending opening hours and providing support in more than one language were suggested directly by participants and respond to barriers that participants described; they are relatively low-cost organisational changes, although their effects have not been evaluated in this setting. Providing information about counsellors’ qualifications and approaches reflects the authors’ interpretation of participants’ uncertainty about counselling, rather than evidence of a training deficit. Psychological screening at admission was suggested by one participant only; screening raises ethical, resource and follow-up questions, would require adequate referral pathways, and its value in this setting would need to be established through broader intervention evidence.

The call for digital booking systems and extended service hours reflects students’ need for greater flexibility within demanding academic schedules. Prior research suggests that accessibility and service responsiveness are important for engagement with mental health support [42,44]. In the current study, participants interpreted delays and rigid appointment structures not merely as logistical inconveniences, but as indicators of limited institutional prioritization of mental health.

Participants’ uncertainty about counsellors’ qualifications and approaches highlights the importance of professional credibility in shaping trust and sustained engagement. Evidence suggests that clarity regarding therapeutic approaches and qualifications enhances perceived service legitimacy [45,46], and similar expectations were articulated by students in this study.

Finally, the emphasis on multilingual provision underscores the importance of culturally and linguistically responsive care. In multilingual societies, language-congruent services have been associated with improved communication and retention in care [36,42]. Within Kazakhstan’s higher education system, linguistic inclusivity may therefore represent a structural component of equitable access to psychological support.

## 5. Limitations and Future Research

Several limitations should be considered when interpreting the findings of this study. First, and most importantly, eligibility required awareness of campus mental health services. Students who were entirely unaware of these services were therefore not included, although limited visibility emerged as a central finding. Our findings on visibility therefore describe students who already knew that services existed and are likely to underestimate the extent of non-awareness.

Second, the sample was small and heterogeneous: 11 students from five universities, with one to four participants per institution, only three men, and only six participants who had used services. Accounts of individual institutions, differences between men and women, and assessments of service quality therefore rest on very few participants and should not be read as institutional, gender-based or sector-level comparisons. All participants studied in a single city, and perspectives from other regions of Kazakhstan may reveal additional institutional or cultural variations.

Third, the study focused exclusively on students’ perspectives. While this approach provided important insight into lived experiences of service accessibility and quality, the inclusion of counselors, university administrators, and policy-makers could offer a more comprehensive understanding of institutional structures and decision-making processes.

Fourth, as with all qualitative research, interpretation of the data may be influenced by researcher reflexivity. Efforts were made to enhance rigor through systematic coding procedures, audit trails, and ongoing reflection; however, complete elimination of interpretive influence is not possible.

Fifth, participants were recruited through open invitations and volunteered to take part, which may introduce self-selection bias; participants also varied in their relationship to the services (used, attempted, or aware only), and only a few participants represented each university, which limits comparison. Interviews were conducted in more than one language and in two modes (face-to-face and zoom), and were translated into English for analysis, in addition the differences in mode may have shaped expression, and altered culturally specific meanings and influenced theme development. Recall bias is also possible. Social desirability may also have affected responses on sensitive topics such as stigma and institutional criticism.

Sixth, participants’ reports of waiting times, staffing, language provision, counsellors’ qualifications and differences between institutions were not corroborated by administrative data, service records, document review or interviews with service providers, and should be read as participants’ perceptions.

Future research would benefit from multi-site studies across diverse regions of Kazakhstan, as well as mixed-methods designs combining qualitative insights with quantitative assessment of service utilization patterns. Longitudinal research may also help examine how institutional reforms, digital innovations, and stigma-reduction initiatives influence help-seeking behavior over time.

## 6. Conclusions

This exploratory study suggests that students’ engagement with university mental health services in Astana is shaped by the interaction of psychological distress, institutional organization, and sociocultural norms. While academic pressure and emotional strain motivated help-seeking, whether students actually used services appeared to depend largely on structural accessibility, service visibility, and perceived legitimacy of counseling within the university environment. Beyond commonly identified barriers such as stigma and logistical constraints, participants described context-specific dynamics, including limited visibility of services in public universities, intergenerational concerns surrounding mental health labeling, and linguistic barriers in counseling provision. These factors suggest that strengthening campus-based mental health systems requires not only expanding service capacity but also addressing culturally embedded and institutionally shaped obstacles to help-seeking.

By foregrounding students’ perspectives, this study contributes empirical evidence from Astana, Kazakhstan. Given the small, single-city sample, these findings are best understood as a starting point for larger, multi-stakeholder research rather than as conclusions generalizable to Kazakhstani higher education as a whole.

## Data Availability

The datasets generated during and/or analyzed during the current study are available from the corresponding author upon request.

## Supplementary Materials

The following supporting information can be downloaded at https://www.mdpi.com/article/10.3390/healthcare14213674/s1. Supplementary Material S1: Interview guide; Supplementary Material S2: Detailed description of the codebook; Supplementary Material S3: Completed Consolidated Criteria for Reporting Qualitative Research (COREQ) checklist.

## References

[1] Kessler R.C., Berglund P., Demler O., Jin R., Merikangas K.R., Walters E.E. (2005). Lifetime prevalence and age-of-onset distributions of DSM-IV disorders in the National Comorbidity Survey Replication. Arch. Gen. Psychiatry.

[2] Lipson S.K., Zhou S., Abelson S., Heinze J., Jirsa M., Morigney J., Patterson A., Singh M., Eisenberg D. (2022). Trends in college student mental health and help-seeking by race/ethnicity: Findings from the national Healthy Minds Study, 2013–2021. J. Affect. Disord..

[3] Auerbach R.P., Mortier P., Bruffaerts R., Alonso J., Benjet C., Cuijpers P., Demyttenaere K., Ebert D.D., Green J.G., Hasking P. (2018). WHO World Mental Health Surveys International College Student Project: Prevalence and distribution of mental disorders. J. Abnorm. Psychol..

[4] Aliev A.-A., Roberts T., Magzumova S., Panteleeva L., Yeshimbetova S., Krupchanka D., Sartorius N., Thornicroft G., Winkler P. (2021). Widespread collapse, glimpses of revival: A scoping review of mental health policy and service development in Central Asia. Soc. Psychiatry Psychiatr. Epidemiol..

[5] Kagarmanova A., Collins N. (2019). The prevalence of depression among Nazarbayev University students and the use of psychological counselling on campus. J. Health Dev..

[6] Gulliver A., Griffiths K.M., Christensen H. (2010). Perceived barriers and facilitators to mental health help-seeking in young people: A systematic review. BMC Psychiatry.

[7] Radez J., Reardon T., Creswell C., Lawrence P.J., Evdoka-Burton G., Waite P. (2021). Why do children and adolescents (not) seek and access professional help for their mental health problems? A systematic review. Eur. Child Adolesc. Psychiatry.

[8] Barrow E., Thomas G. (2022). Exploring perceived barriers and facilitators to mental health help-seeking in adolescents: A systematic literature review. Educ. Psychol. Pract..

[9] Lui J.C., Sagar-Ouriaghli I., Brown J.S.L. (2024). Barriers and facilitators to help-seeking for common mental disorders among university students: A systematic review. J. Am. Coll. Health.

[10] Abzaliyeva D., Umurkulova M., Duisekova S., Mussatay G. (2023). Relevance of the mental health problem of higher education students: Analysis and prospects of solution for Kazakhstan. J. Health Dev..

[11] Hook K., Bogdanov S. (2021). Mental health care in Eastern Europe and Central Asia: An analysis of needs and a call for greater investment. Lancet Reg. Health Eur..

[12] Hundley G., Lambie G.W. (2007). Russian speaking immigrants from the Commonwealth of Independent States in the United States: Implications for mental health counselors. J. Ment. Health Couns..

[13] Cavanagh D., Jurcik T., Charkhabi M. (2021). How does trust affect help-seeking for depression in Russia and Australia?. Int. J. Soc. Psychiatry.

[14] Hansen A., McGarry D., Johnson A., Roche M.A. (2021). The impact of an undergraduate students’ culture on their learning about mental health: A scoping review. Nurs. Health Sci..

[15] Hernández-Torrano D., Ibrayeva L., Sparks J., Lim N., Clementi A., Almukhambetova A., Nurtayev Y., Muratkyzy A. (2020). Mental health and well-being of university students: A bibliometric mapping of the literature. Front. Psychol..

[16] Jorm A.F. (2000). Mental health literacy: Public knowledge and beliefs about mental disorders. Br. J. Psychiatry.

[17] Angermeyer M.C., Dietrich S. (2006). Public beliefs about and attitudes towards people with mental illness: A review of population studies. Acta Psychiatr. Scand..

[18] Republic of Kazakhstan (2020). Code of the Republic of Kazakhstan No. 360-VI “On Public Health and Healthcare System.” Adilet Legal Information System. https://adilet.zan.kz/eng/docs/K2000000360.

[19] Ministry of Health of the Republic of Kazakhstan (2020). Order No. KR DSM-203/2020 “On Certain Issues of Providing Medical and Social Assistance in the Field of Mental Health” (as Amended). Adilet Legal Information System.

[20] Order of the Minister of Health and Social Development of the Republic of Kazakhstan No. 188. (31 March 2015) “On Approval of the List of Medical Psychiatric Contraindications for Engaging in Certain Types of Professional Activities, as well as Work Involving Sources of Increased Danger”. https://adilet.zan.kz/rus/docs/V1500010858.

[21] Article 11 of the Code of the Republic of Kazakhstan “On Marriage (Matrimony) and Family”. https://adilet.zan.kz/eng/docs/K1100000518.

[22] Lau J.Y., Murray A., Palauskaite L., Aidralieva N., Beknazarova M., Ismailova L., Matvienko I., Nigmadjanova G., Osmonalieva M., Panteleeva L. (2026). Youth mental health in Central Asia: Research needs. Lancet Psychiatry.

[23] UNICEF (2018). Study on Prevalence, Underlying Causes, Risk and Protective Factors in Respect to Suicides and Attempted Suicides in Kazakhstan.

[24] UNICEF Kazakhstan (2025). Country Office Annual Report 2024.

[25] Creswell J.W., Poth C.N. (2018). Qualitative Inquiry and Research Design: Choosing Among Five Approaches.

[26] Patton M.Q. (2015). Qualitative Research & Evaluation Methods.

[27] Malterud K., Siersma V.D., Guassora A.D. (2016). Sample size in qualitative interview studies: Guided by information power. Qual. Health Res..

[28] Mrazek P.J., Haggerty R.J., Institute of Medicine (US) Committee on Prevention of Mental Disorders (1994). Reducing Risks for Mental Disorders: Frontiers for Preventive Intervention Research.

[29] Barry M.M., Clarke A.M., Petersen I., Jenkins R., Quah S.R., Cockerham W.C. (2019). Implementing mental health promotion. Springer Handbook of Public Health.

[30] Braun V., Clarke V. (2021). One size fits all? What counts as quality practice in (reflexive) thematic analysis?. Qual. Res. Psychol..

[31] Fereday J., Muir-Cochrane E. (2006). Demonstrating rigor using thematic analysis: A hybrid approach of inductive and deductive coding and theme development. Int. J. Qual. Methods.

[32] Lincoln Y.S., Guba E.G. (1985). Naturalistic Inquiry.

[33] Tong A., Sainsbury P., Craig J. (2007). Consolidated criteria for reporting qualitative research (COREQ): A 32-item checklist for interviews and focus groups. Int. J. Qual. Health Care.

[34] Hyseni Duraku Z., Davis H., Hamiti E. (2023). Mental health, study skills, social support, and barriers to seeking psychological help among university students: A call for mental health support in higher education. Front. Public Health.

[35] Alvarez-Hernandez L.R., Childs E.M., Fatehi M., Yeo H. (2024). How perception relates to student utilization of college campus counseling services. J. Am. Coll. Health.

[36] King N., Pickett W., Pankow K., Dimitropoulos G., Cullen E., McNevin S., Patten S.B., Duffy A. (2024). Access to university mental health services: Understanding the student experience. Can. J. Psychiatry.

[37] Akhmetsadyk Z., Mynbayeva A., Kudaibergenova A. (2023). University psychological support services in Almaty. J. Psychol. Sociol..

[38] Trusty W.T., Scofield B.E., Janis R.A., Cummins A.L., White T.D. (2026). Psychotherapy dose, clinical outcome, and academic withdrawal at university counseling centers. Psychol. Serv..

[39] Janota M., Kovess-Masfety V., Gobin-Bourdet C., Husky M. (2022). Use of mental health services and perceived barriers to access services among college students with suicidal ideation. J. Behav. Cogn. Ther..

[40] Milanzi J., Kabinga Makukula M., Lyambai K., Kabungo C.J., Matipa R., Changwe G. (2023). Barriers to mental health seeking intentions among students at Mulungushi University, Kabwe District, Zambia. Eur. J. Public Health Stud..

[41] Seidler Z.E., Rice S.M., Kealy D., Oliffe J.L., Ogrodniczuk J.S. (2020). What gets in the way? Men’s perspectives of barriers to mental health services. Int. J. Soc. Psychiatry.

[42] Ghaffar D., Berry C., Tunks A., Hazell C.M., Niven J.E. (2024). A thematic analytic account of university support services’ acceptability for postgraduate researchers experiencing mental health problems. Couns. Psychother. Res..

[43] Yorgason J.B., Linville D., Zitzman B. (2008). Mental health among college students: Do those who need services know about and use them?. J. Am. Coll. Health.

[44] Johnson R.L., Nandan M., Culp B., Thomas D. (2023). College students’ mental health help-seeking behaviors. Coll. Stud. Aff. J..

[45] Pedrelli P., Nyer M., Yeung A., Zulauf C., Wilens T. (2015). College students: Mental health problems and treatment considerations. Acad. Psychiatry.

[46] National Academies of Sciences, Engineering, and Medicine (2021). Mental Health, Substance Use, and Wellbeing in Higher Education: Supporting the Whole Student.

