# Supplementary material for: Barriers and Facilitators to Using Campus-Based Mental Health Services: An Exploratory Qualitative Study of University Students in Astana, Kazakhstan
Source: Healthcare (Basel). 2026 Oct 29;14(21):3674. doi: 10.3390/healthcare14213674 (PMC13762565; doi:10.3390/healthcare14213674)
Supplement: Supplementary file 1 [file healthcare-14-03674-s001.zip › healthcare-4545665-supplementary S1 and S2.pdf]

S1. Interview guide

Thank you for participating in this interview. It should take about 30–60 minutes. I'm here to learn about your experiences, attitudes, and approaches regarding the psychological support available to university students, including how mental health services are utilized, perceived, and experienced within your university.

I. Opening

1. Can you start by sharing a bit about your background and what year you are in at university?
    - **Probing Questions:**
        - What do you study, and how has your university experience been so far?
        - Has your academic environment impacted your mental well-being?

II. Mental Health Services Overview

2. Are you aware of any mental health services available at your university? What do you think these services focus on when supporting students' mental health?
    - **Probing Questions:**
        - Do you think these services prioritize prevention, treatment, or maintenance? Why?
        - Can you provide specific examples of how these areas are addressed?
        - Which of these areas do you think is most needed at your university?
3. **How supportive do you feel your university environment is regarding mental health?**
    - **Probing Questions:**
        - Are there specific spaces, faculty, or staff you find supportive?
        - Does your university encourage open discussions about mental health?

III. Utilization of Mental Health Services

4. Have you used or considered using mental health services at your university? Why or why not?
    - **Probing Questions:**
        - What factors influenced your decision to use them, or what held you back?
        - Were there specific moments or needs that led you to this decision?
5. What factors have influenced your decision to seek or not seek help?
    - **Probing Questions:**

- Have you ever felt hesitant to seek help because of stigma?
- How do you think these factors impact other students?

IV. Accessibility and Quality

6. How accessible do you find the mental health services at your university?

    - **Probing Questions:**
        - Can you describe the ease of accessing these services (e.g., location, affordability, or wait times)?
        - Have these factors ever discouraged you or others from using these services?

7. **If you have used these services, how would you describe your experience with their quality?**

    - **Probing Questions:**
        - How would you evaluate the skills or empathy of the counselors?
        - Do you feel cultural or individual student needs are adequately addressed?

V. Cultural and Social Influences

8. Are there any cultural or social factors that you think affect students' willingness to seek mental health support?
    - **Probing Questions:**
        - How do you think mental health is perceived in your community or family?
        - Do you feel there is pressure to "handle things on your own" instead of seeking help?

VI. Supportive Environments and Improvements

9. What are your thoughts on how mental health support for students in Kazakhstan could be improved?
    - **Probing Questions:**
        - If you could change or add one thing to your university's mental health services, what would it be?
        - What role do you think universities or the government should play in addressing mental health challenges?

VII. Closing Questions

10. Is there anything else you'd like to share about your experiences or thoughts on mental health services?
    - **Probing Questions:**
        - Do you think there's an important issue we didn't cover?

Thank you for your time and insights. Your contribution is greatly appreciated and will help improve our understanding of mental health support for university students.

S2. Codebook for a qualitative study

Research questions:

(1) What factors influence students' utilization of campus-based mental health services?;

(2) How do students perceive the accessibility and quality of available mental health support services?

(3) What recommendations can be derived from students' experiences to enhance university mental health services?

Unit of analysis: interview transcripts

Descriptors:

1. Gender (male & female)
2. Year of study (freshmen, sophomore, junior, senior)
3. Type of university (private & public)

Code	Description	Example
**1. Awareness of personal psychological issues**	Interviewees noted recognizing their mental health struggles, which prompted them to seek help.	*"My grades became very bad... my sleep was disrupted." [PW3, junior, private]*
**2. Accessibility of services**	Participants share their experience of the availability and ease of access to mental health services on campus.	

a. Ease and convenience	Interviewee mentioned the availability of multiple contact methods for scheduling, making access easier.	*"You can just write to WhatsApp or call, and you can come at any time." [PM2, sophomore, public]*
b. Campus location and physical proximity	Students highlighted the convenience of having mental health services located near campus facilities like dormitories.	*"Our university is well structured, easy access... you can get help quickly." [PW1, senior, private]*
c. Lack of immediate appointments	Students shared frustration about delays in securing urgent mental health appointments, causing them to seek outside services.	*"At university, they signed me up for a certain date; there was no urgent appointment." [PW4, sophomore]*

**3. Social and cultural influences**	Interviewees talk about societal and cultural factors that impact students' willingness to access mental health services, such as stigma and familial attitudes.	
a. Stigma and societal perceptions	Students spoke about fear of judgment and common misconceptions about mental health services.	*"People from rural areas still believe psychologists are only for mentally ill." [PM2, sophomore, public]*

b. Influence of family attitudes	Students described how family members' lack of understanding or concern about mental health influenced their decisions to seek help.	*"My parents thought it was unnecessary, even dangerous." [PW3, junior*]
c. Masculinity norms	Male students mentioned the reluctance to seek help due to societal expectations around masculinity.	*"It's taboo among guys; they delay asking for help simply because 'I'm a man'." [PW3, junior, private1]*

**4. Perceptions of service quality**	Students share their evaluations of the quality of mental health services, including their satisfaction and concerns about professionalism.	
a. Positive accessibility experiences	Students expressed satisfaction with easy access to mental health services and appointments.	*"You can easily make an appointment; it's free." [PW4, sophomore, private1]*

**b.** Negative experiences due to system inefficiencies	Students reported frustration with delays and scheduling inefficiencies, which led to dissatisfaction.	"The next appointment will be in a month; the problem will become outdated by then." [PW6, junior, private2]
c. Concerns regarding professionalism and competence	Students questioned whether counselors were adequately trained and experienced, impacting their trust in services.	*"I'm not sure the woman was really a psychologist... I wasn't 100% sure she knew what she was doing." [PW4, sophomore, private1]*

d. Lack of personalized or appropriate therapeutic approaches<br><br>e. Mixed experiences of psychological support	Students felt that the therapeutic methods used were not individualized or effective for their needs.	"The techniques felt vague; after all I shared, I got a simple meditation exercise." [PW4, sophomore, private1]
	Students shared both positive and negative experiences with the psychological support they received.	*"My psychologist was professional and supportive; I could trust her." [PW3, junior, private1]*

**5. Recommendations for improvement**	Interviewees suggestions for improving mental health services on campus.	
a. Enhanced awareness and visibility	Students suggested promoting mental health services across the campus to raise awareness.	*"I would never have known if I didn't live in a dorm." [PM2, sophomore, public1]*<br><br>*"Workshops or stress-relief weeks can encourage students to share openly." [PW3, junior, private1]*
b. Improving service accessibility	Suggestions included digital solutions for easier appointment scheduling.	"It would be better to use a bot or app for appointments." [PW4, sophomore, private1]
c. Strengthening professional competence	Students recommended better transparency regarding the qualifications of counselors.	*"We should have more information about counselors' backgrounds and certifications." [PW1, senior, private1]*
d. Addressing language and cultural diversity	Students emphasized offering multilingual support to accommodate diverse backgrounds.	*"Understanding each other is essential; otherwise, conversations are useless." [PW1, senior, private1]*
