# Supplementary material for: Barriers and Facilitators to Using Campus-Based Mental Health Services: An Exploratory Qualitative Study of University Students in Astana, Kazakhstan
Source: Healthcare (Basel). 2026 Oct 29;14(21):3674. doi: 10.3390/healthcare14213674 (PMC13762565; doi:10.3390/healthcare14213674)
Supplement: Supplementary file 1 [file healthcare-14-03674-s001.zip › healthcare-4545665-supplementary S3.pdf]

Supplementary Material S3. COREQ Checklist

Consolidated criteria for reporting qualitative research (Tong et al., 2007). Items highlighted in yellow must be completed by the co-authors before submission.

No.	Item	Response	Section
**Domain 1: Research team and reflexivity**			
1	Interviewer/facilitator	All interviews were conducted by the first author (I.S.), Supervisors (Y.N., K.A.Y.) supported analysis and interpretation process.	2.5
2	Credentials	MPH.	2.5
3	Occupation	Public health researcher.	2.6
4	Gender	Female.	2.5
5	Experience and training	Trained in qualitative interviewing; supervised by the senior authors (K.A.Y., Y.N.), who provided ongoing debriefing.	2.5
6	Relationship established	No prior relationship with participants; recruitment was through open invitations.	2.5
7	Participant knowledge of the interviewer	Participants had no prior knowledge of the interviewer before taking part in the study.	2.3, 2.5
8	Interviewer characteristics	Female, MPH-trained researcher familiar with the university context; positionality and assumptions were discussed within the team.	2.6
**Domain 2: Study design**			
9	Methodological orientation and theory	Framework-informed qualitative descriptive study (Modified Mental Health Intervention Spectrum); codebook thematic analysis with a hybrid deductive–inductive approach.	2.1, 2.2, 2.6
10	Sampling	Purposive sampling.	2.3
11	Method of approach	Open invitations through student group chats and social media; interested students were contacted by the first author	2.3
12	Sample size	11 participants.	2.3; Table 1
13	Non-participation	15 responded; 2 not screened; 13 screened and eligible; [1] declined and [1] could not be scheduled.	2.3
14	Setting of data collection	Private rooms on university campuses (n = 5) or Zoom (n = 6).	2.5
15	Presence of non-participants	None.	2.5
16	Description of sample	Gender, year of study, institution type, age, field of study, university, service-use status, interview language and mode, and residence.	Table 1
17	Interview guide	10 open-ended questions informed by the framework; pilot-tested; provided as Supplementary Material 1.	2.5
18	Repeat interviews	None.	2.6
19	Audio/visual recording	Audio-recorded.	2.5
20	Field notes	Taken by the first author (I.S.),	2.6
21	Duration	30–60 minutes.	2.5

Public

No.	Item	Response	Section
22	Data saturation	Code saturation assessed across the whole sample; information power used as a complementary rationale.	2.3
23	Transcripts returned	No.	2.6
**Domain 3: Analysis and findings**			
24	Number of data coders	More than one: by the first author (I.S.), and Supervisors (Y.N., K.A.Y.)	2.6
25	Description of the coding tree	Codebook provided as Supplementary Material 2.	2.6
26	Derivation of themes	Hybrid: initial codes derived deductively from the framework and interview guide; further codes generated inductively from the data.	2.2, 2.6
27	Software	NVivo.	2.6
28	Participant checking	Not undertaken.	2.6
29	Quotations presented	Yes; each identified by participant code, year of study, university and service-use status.	3
30	Data and findings consistent	Yes; frequencies are reported as numbers of participants.	3, 4
31	Clarity of major themes	Five themes, presented in Figure 1 and Table 2.	3
32	Clarity of minor themes	Subthemes are reported within each theme; contrasting cases are described.	3
